# Effect of a Dipeptidyl Peptidase-IV Inhibitor, Des-Fluoro-Sitagliptin, on Neointimal Formation after Balloon Injury in Rats

**DOI:** 10.1371/journal.pone.0035007

**Published:** 2012-04-06

**Authors:** Soo Lim, Sung Hee Choi, Hayley Shin, Bong Jun Cho, Ho Seon Park, Byung Yong Ahn, Seon Mee Kang, Ji Won Yoon, Hak Chul Jang, Young-Bum Kim, Kyong Soo Park

**Affiliations:** 1 Department of Internal Medicine, Seoul National University Bundang Hospital, Seongnam, Korea; 2 Department of Internal Medicine, Seoul National University College of Medicine, Seoul, Korea; 3 Johns Hopkins Bloomberg School of Public Health, Baltimore, Maryland, United States of America; 4 Division of Endocrinology, Diabetes and Metabolism, Department of Medicine, Beth Israel Deaconess Medical Center and Harvard Medical School, Boston, Massachusetts, United States of America; 5 Department of Molecular Medicine and Biopharmaceutical Sciences, Graduate School of Convergence Science and Technology and College of Medicine, Seoul National University, Seoul, Korea; Kaohsiung Chang Gung Memorial Hospital, Taiwan

## Abstract

**Background:**

Recently, it has been suggested that enhancement of incretin effect improves cardiac function. We investigated the effect of a DPP-IV inhibitor, des-fluoro-sitagliptin, in reducing occurrence of restenosis in carotid artery in response to balloon injury and the related mechanisms.

**Methods and Findings:**

Otsuka Long-Evans Tokushima Fatty rats were grouped into four: control (normal saline) and sitagliptin 100, 250 and 500 mg/kg per day (*n* = 10 per group). Sitagliptin or normal saline were given orally from 1 week before to 2 weeks after carotid injury. After 3 weeks of treatment, sitagliptin treatment caused a significant and dose-dependent reduction in intima-media ratio (IMR) in obese diabetic rats. This effect was accompanied by improved glucose homeostasis, decreased circulating levels of high-sensitivity C-reactive protein (hsCRP) and increased adiponectin level. Moreover, decreased IMR was correlated significantly with reduced hsCRP, tumor necrosis factor-α and monocyte chemoattractant protein-1 levels and plasminogen activator inhibitor-1 activity. In vitro evidence with vascular smooth muscle cells (VSMCs) demonstrated that proliferation and migration were decreased significantly after sitagliptin treatment. In addition, sitagliptin increased caspase-3 activity and decreased monocyte adhesion and NFκB activation in VSMCs.

**Conclusions:**

Sitagliptin has protective properties against restenosis after carotid injury and therapeutic implications for treating macrovascular complications of diabetes.

## Introduction

A novel approach in the treatment of type 2 diabetes is to harvest the actions of incretin hormones such as glucagon like peptide (GLP)-1 and glucose-dependent insulinotropic peptide (GIP). GLP-1 and GIP are gut peptides and are secreted in a nutrient-dependent manner, stimulating insulin secretion glucose-dependently. In rodents, these peptides promote pancreatic beta cell proliferation and inhibit apoptosis, leading to expansion of beta cell mass [1], [2]. Further, GLP-1 improves glucose homeostasis via additional actions on both glucose sensors, inhibition of gastric emptying, food intake and glucagon secretion [3]. As such, GLP-1 administration potently stimulates insulin secretion and reduces blood glucose level in human subjects with type 2 diabetes [4].

Dipeptidyl peptidase IV (DPP-IV) is the enzyme responsible for the degradation of endogenous GLP-1 and GIP, rapidly cleaving and inactivating GLP-1 and GIP into inactive metabolites [5]. Hence, one recent glucose-lowering strategic approach is to enhance active incretin hormone levels and activity through the development of selective DPP-IV inhibitors [6], [7]. The approach in both preclinical animal models of type 2 diabetes and in clinical studies of patients with type 2 diabetes has shown to increase levels of intact GLP-1 and GIP, ultimately leading to meaningful improvements in overall glucose homeostasis [8]–[10].

Cardiovascular disease is the main cause of death in type 2 diabetes. Almost 80% of diabetes patients die from cardiovascular diseases such as myocardial infarction or stroke. Primary coronary intervention with stent implantation is now widely performed to patients with symptomatic coronary artery diseases. Although the development of drug eluting stents have reduced the incidence of restenosis after coronary intervention, restenosis of the intervened vessel is still a critical issue of significant magnitude [11].

Interestingly, a recent paper proved that augmentation of GLP-1 by inhibition of DPP-IV improved left ventricle performance in response to stress in patients with coronary artery disease [12]. There is evidence suggesting effects of GLP-1 on various cardiovascular risk factors [13]. However, whether DPP-IV inhibition has a role in preventing restenosis after vascular injury and more generally has potential anti-atherosclerotic properties has not been adequately explored.

Plasma levels of matrix metalloproteinase (MMP) 2 and 9 are increased in diabetic patients reflecting changes in extracellular matrix (ECM) metabolism [14]. The expression and activity of MMPs in diabetes thus far have been reported to correlate with the macrovascular and microvascular complications seen in this disease [15], [16]. However, there have been no data available about the role of DPP-IV inhibitor on MMPs in type 2 diabetic patients.

Since the DPP-IV inhibitors have been shown to improve glucose homeostasis, their profile relating to atherosclerosis should also be elucidated. In relation to this, some of the cytokines generated from fat cells, known as “adipocytokines", have been reported to be associated with chronic diabetic complications [17], [18]. A central adipokine is adiponectin, the lower concentration of which is reported to increase risks of macrovascular complications [17], [19]. Until now, there have been limited reports on adiponectin changes following treatment with DPP-IV inhibitors. In addition, it has been reported that DPP-IV interacting with caspase recruitment domain family, member 11 (CARD11 or CARMA1) leads to NFκB activation in T-cells [20]. NFκB is a common regulator that involves in the control of proinflammatory genes and vascular smooth muscle cells proliferation.

In the present study, we investigated the ability of a DPP-IV inhibitor, des-fluoro-sitagliptin, in reducing occurrence of restenosis after vascular balloon injury and the related mechanisms by applying a model of obese animals with naturally developing type 2 diabetes and vascular cell lines.

## Methods

### Study Animals and Care for the Animals

Forty Otsuka Long-Evans Tokushima Fatty (OLETF) rats (5 week old, male), the animal model for obese, type 2 diabetes, were donated by the Japanese Otsuka Pharmaceuticals. OLETF rats naturally develop type 2 diabetes around 24 weeks of age and have been applied in studies investigating glucose metabolism and cardiovascular complications [21]. A DPP-IV inhibitor des-fluoro-sitagliptin (Merck, NJ, USA) was administered to twenty-four weeks old OLETF rats to test the hypothesis that sitagliptin can reduce the restenosis in carotid artery after balloon injury. Rats were grouped into four groups: 1) Control (saline), 2) Sita100 (sitagliptin 100 mg/kg per day), 3) Sita250 (sitagliptin 250 mg/kg per day), and 4) Sita500 (sitagliptin 500 mg/kg per day) (*n*  =  10 per group). Sitagliptin or normal saline were given orally for 1 week before and 2 weeks after carotid injury by using an oral Zonde needle (Natsume, Tokyo, Japan).

Another 20 Long-Evans Tokushima Otsuka (LETO) rats, a normal counterpart of the OLETF rat, were also donated by the Otsuka Pharmaceutical Co. (Tokushima, Japan). Sitagliptin (500 mg/day) or normal saline was administered to the rats (n  =  10 in each group) for 3 weeks to test the same hypothesis in this normal control animal.

Animals were handled in compliance with the Guide for Experimental Animal Research from the Laboratory for Experimental Animal Research, Clinical Research Institute, Seoul National University Hospital. Seoul National University Hospital Ethics Committee for Animal Study approved this study (07177).

### Animal Study

**(1) Active GLP-1 and glucagon levels in plasma of study rats.** After 3 weeks treatment of sitagliptin, plasma active GLP-1 levels were measured by an enzyme immunoassay (RENDO-85K, Linco Research, St. Charles, MO, USA). After collecting blood samples, tube was inverted several times to mix, and 10 µL of DPP-IV inhibitor (Millipore) per every ml of blood collected was added immediately. Then the blood was centrifugated for 10 minutes at 1000 × g after clotting for 30 minutes. Plasma glucagon level was measured by the same kit (RENDO-85K, Linco Research).

**(2) Rat carotid artery balloon denudation injury.** A previously well-established rat carotid artery balloon injury model was used in this study [22]. Rats were anesthetized with a combination anesthetic (ketamine, 70 mg/kg; xylazine, 7 mg/kg IP; Yuhan Corp, Seoul, Korea). After the left external carotid artery was exposed, heparin (35 IU) is administered systemically via the external jugular vein. A 2F Fogarty embolectomy catheter (Baxter Healthcare Corp, IL, USA) was introduced into an external carotid arteriotomy incision, advanced to the common carotid artery, and inflated with 0.2 mL of saline and withdrawn 10 times with rotation.

**(3) Morphometric analysis.** Two weeks after balloon injury, rats were euthanized with a lethal dose of pentobarbital, and carotid arteries were fixed by perfusion at 120 mmHg with 4% formaldehyde via an 18G intravenous cannula placed retrograde in the abdominal aorta. Tissues were then embedded in paraffin, and sections were stained with H&E. The extent of neointimal formation in histologically stained sections was quantified by computed planimetry. The cross-sectional areas of the blood vessel layers, ie, the lumen, intimal, and medial areas, are quantified in 3 different sections (proximal, middle, and distal) using an Image-Pro Plus Analyzer Version 4.5 (Media Cybernetics, MD, USA). The intima-media ratio (IMR) was calculated from the mean of these determinations.

**(4) Immunoblot analysis for DPP-IV and GLP-1 receptors in carotid artery.** Harvested vessel tissues were homogenized with cell lysis buffer (Cell signaling, Beverly, MA, USA) containing 20 mM Tris (pH 7.5), 150 mM NaCl, 1 mM Na_2_EDTA, 1% Triton, 2.5 mM sodium pyrophate, 1 mM β-glycerophosphate, 1 mM Na_3_VO_4_, 1 mg/mL leupeptin, and 1 mM PMSF for 30 minutes at 4°C and protein lysate concentrations were measured by Bradford protein assay kit (BioRad, Hercules, CA, USA). The same amounts of proteins from whole cell lysates were subjected to sodium dodecyl sulfate polyacrylamide gel electrophoresis (SDS-PAGE) and transfer onto methanol-treated PVDF membranes (Millipore Co, Bedford, MA). After blocking the membrane with Tris-buffered saline-Tween 20 (TBS-T, 0.1% Tween 20) containing 5% blocking buffer for 1 hour at room temperature, they were washed with TBS-T and incubated primary antibodies, DPP-IV (Santa Cruz, CA), GLP-1-receptor (Santa Cruz) and γ-tubulin (Sigma, St Louis, MO) for 1 hour at room temperature or for overnight at 4°C. The membranes were washed three times with TBS-T for 10 minutes, and then incubated for 1 hour at room temperature with horseradish peroxidase (HRP)-conjugated secondary antibodies. After extensive washing, the bands were detected by enhanced chemiluminescence (ECL) reagent (Santa Cruz).

**(5) Immunohistochemical staining for proliferation.** To detect proliferating cells, immunohistochemical staining against proliferating cell nuclear antigen (PCNA) were performed on balloon injured arteries. Briefly, paraffin-embedded samples were sectioned and treated with protease K for 4 minutes, and then endogenous peroxidase was quenched with methanol/peroxidase solution. Specimens were treated with 50 mmol/L Tris HCl (pH 7.6) containing 0.15 mol/L NaCl and 0.1% Tween 20 for 5 minutes, and then incubated in 1:50 diluted anti-PC-10 antibody (Dako, Cincinnati, OH) for PCNA staining. Specimens were then processed by incubation with 1:50 diluted 3,39-diaminobenzidine tetrahydrochloride substrate solution (Dako) and counterstained with Mayer hematoxylin (Dako). Proliferation was defined as the percentage of PCNA positive cells versus total nucleated cells in 4 different sectors per tissue section.

**(6) TUNEL staining.** Detection of apoptotic cells *in vivo* was also performed using the TUNEL method with minor modification [23]. Briefly, 5µm sections were deparaffinized and incubated with proteinase K (Dako) (20 µg/mL) for 15 minutes at room temperature. An apoptosis detection kit (Apoptag, Intergen Company) was used with the chromogen DAB. Counterstaining was done with Mayor hematoxylin (Dako). Apoptotic cells were quantified by determining percentages of TUNEL-positive cells versus total nucleated cell count in 4 different sectors per tissue section.

**(7) Immunofluorescence double staining**. Immunofluorescence double staining was performed to localize smooth muscle cells and MMP2-expressing cells by the use of fluorescence-conjugated antibodies in rat to clarify whether MMP2-positive cells co-localize on proliferating smooth muscle cells using primary antibody (rabbit anti-αSMA antibody, 1:50, Dako) and second antibody (anti-rabbit antibody, 1:50, Dako) for smooth muscle cells and primary antibody (mouse anti-MMP2 antibody, 1:250, Santa Cruz) and second antibody (anti-mouse antibody, 1:50, Dako) for MMP2. Immunofluorescence double staining was also performed to localize αSMA (Dako) and MMP9 (Dako) to clarify whether proliferating smooth muscle cells correspond to cells expressing MMP9.

**(8) Glucose metabolism, adiponectin and inflammatory status.** Possible relevant factors affecting the degree of neointimal formation, such as glucose homeostasis, adipocytokines and inflammatory status were evaluated. For glucose homeostasis, an intraperitoneal glucose tolerance test (IPGTT) was done at baseline and 3 weeks after DPP-IV inhibitor treatment. After the 12 h fasting glucose concentration had been measured, each animal was injected intraperitoneally with 1.5 g/kg of 50 M glucose solution. Blood samples (about 10 µL) were collected from an incision in the tail at 30, 60, 90 and 120 min after the glucose load. Plasma glucose concentration was measured using reagent strips read in a glucose meter (YSI 2300-STAT, Yellow Springs, OH). The homeostasis model assessment of the insulin resistance (HOMA-IR) and beta cell function (HOMA-B) were calculated using fasting insulin and glucose levels. In addition, the area under the curve for glucose (AUC_glucose_) was calculated using the trapezoid rule for glucose data from 0 to 120 min. Rat adiponectin and high-sensitivity C-reactive protein (hsCRP) were measured by using ELISA kits developed by Adiopogen (Seoul, Korea) and BD Biosciences Pharmingen (Heidelberg, Germany), respectively. Monocyte chemoattractant protein-1 (MCP-1), TNFα and plasminogen activator inhibitor-1 (PAI-1) activity were also measured by a Multiplex kit (RADPK-81K, Linco).

### Cell Study

Rat aortic smooth muscle cells (RAoSMCs) were obtained from Bio-Bud (Seoul, Korea) and cultured in Dulbecco’s modified Eagle’s medium (Gibco BRL, Grand Island, NY) supplemented with 10% fetal bovine serum (FBS) and 100 U/mL penicillin-streptomycin. Primary cultures of human umbilical vein endothelial cells (HUVECs; Cambrex, Walkersville, MD) were cultured in endothelial cell growth medium (EGM-2; Cambrex) containing 2% FBS, 0.4% hydrocoritisone, 4% hFGF-B, 0.1% VEGF, 0.1% R3-IGF, 0.1% ascorbic acid, 0.1% hEGF, 0.1% GA-1000, and 0.1% heparin, according to the manufacturer’s instruction. Cells were grown in an atmosphere of 95% air and 5% CO_2_ at 37°C. RAoSMCs were starved in DMEM supplemented without for 48 hours and used for the experiments. HUVECs were starved in endothelial cell basal medium (EBM-2) (Cambrex) supplemented with 0.2% FBS for 24 hours and used for the experiments. Subcultured RAoSMCs from passages 4 to 11 and HUVECs from passages 2 to 9 were used in this experiment.

**(1) Cell proliferation assessed by MTT and thymidine incorporation assay.** Cell proliferation was determined by a modified 3-(4,5-dimethyl-thiazol-2-yl)-2,5-diphenyltetrazolium bromide (MTT) assay. MTT (Sigma) (5 mg/mL) was dissolved in PBS. RAoSMCs were grown in 48-well plates at a density of 2×10^3^ per well, and starved for 48 hours, and then placed in DMEM supplemented with 0.5% FBS. The cells were exposed to different doses of sitagliptin for 24 and 48 hours. The same method was used for HUVECs. HUVECs were grown in 48-well plates at a density of 2×10^3^ per well. After 24 hours of starvation, EBM-2 was supplemented with 0.2% FBS. The cells were exposed to the same dose of sitagliptin for 24 and 48 hours, after 0.5 mL of MTT solution (5 mg/mL) in starvation medium was added to each well, and the plates were incubated for 4 hours at 37°C in 5% CO_2_/air. The medium was removed carefully, and the purple dye was dissolved in 0.1 mL dimethyl sulfoxide. After 10-minute incubation, 200 µL from each well was transferred to a 96-well plate, and the absorbance was measured at 570 nm with a spectrophotometer.

For the thymidine incorporation assay, VSMCs (5×10^4^ cells) were seeded in 24-well plates in DMEM with 10% FBS. For serum-induced or platelet-derived growth factor (PDGF) stimulation, cells were incubated with 2% fetal bovine serum (FBS) or 10 ng/mL of PDGF (R&D Systems, Camarillo, USA) for 24 h, respectively. Subsequently, [^3^H]-thymidine at 1 µCi per well was incubated for a further 4 h. The incubation was terminated by 5% trichloroacetic acid (TCA) for 20 min. The fixed cells were then washed twice with 100% ethanol and treated with 0.3M NaOH in 2% Na_2_CO_3_. The tritium content of lysates was counted by LS6500 Multipurpose Scintillation Counter (Beckman, CA).

**(2) Cell migration assessed by wound healing assay.** RAoSMCs were grown to confluence in 6-well plates and then starved in DMEM with 0.5% FBS for 48 hours. Each well was divided into a 2×3 grid. Using a 100–1000 µL pipette tip, a linear wound was made in each hemisphere of the well. Immediately after wounding, the medium was changed to starvation medium. All reagents were mixed in starvation medium. Images were taken of the intersections of the linear wound and each grid line, which resulted in 3 fields per well. Cells were allowed to migrate for 24 hours at 37°C. Each field was measured at 24 hours.

**(3) Monocyte adhesion assay.** U937 cells (ATCC, Rockville, MD) were washed 3 times with serum-free RPMI media and then resuspended in this medium. U937 cells (1.25×10^4^) were added to the HUVEC monolayers stimulated with TNFα (10 ng/mL) for 18 hours and incubated for 30 minutes at 37°C in 5% CO_2_. Unbound cells were removed by washing 3 times with PBS. EBM-2 media was then added and only the U937 cells adhering to an endothelial cell were counted in 3–5 randomly selected fields of view in each well by a phase-contrast microscopy.

**(4) Measurement of caspase-3 activity.** 1×10^4^ cells were seeded into microplate and incubated in DMEM with 10% FBS, then starved in DMEM with 0% FBS for 24 hr. For TNFα stimulation, the cells were incubated with 10 ng/mL of TNFα (R&D systems, Camarillo). The cells were treated with 25, 50, 100, and 200 µg/ml of sitagliptin for 24 h. Caspase-3 activity was measured by using Caspase-Glo 3/7 assay kit (Promega, WI, USA).

**Figure 1:**
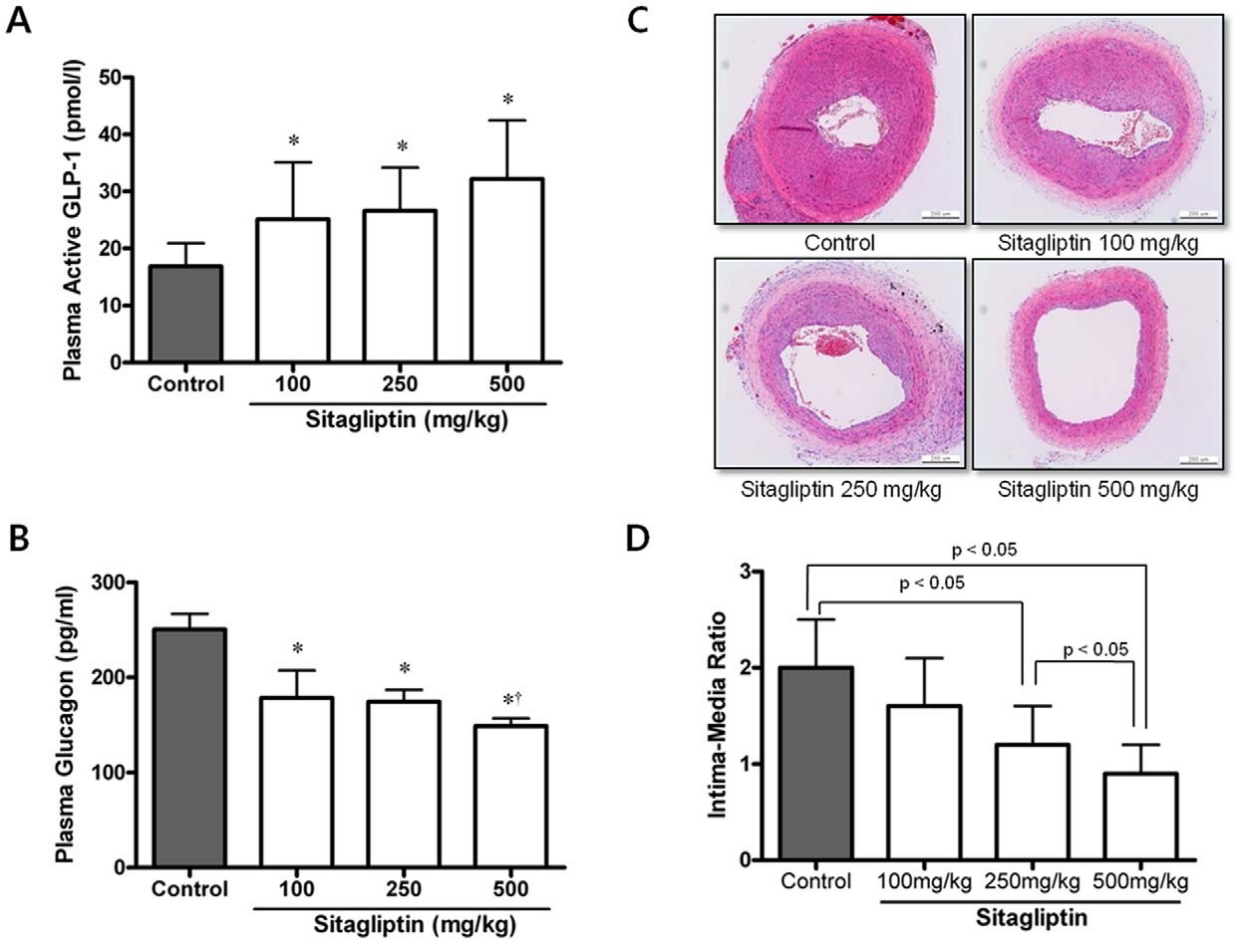
Plasma active GLP-1 (A) and glucagon (B) levels after 3 week treatment of sitagliptin. Plasma active GLP-1 levels increased and glucagon levels decreased significantly in a dose-dependent manner (*p <0.05 compared with control and †p <0.05 compared with 100 mg/kg of sitagliptin treatment). In vivo inhibition of neointimal formation after 3 weeks of treatment with sitagliptin. C. H&E stained sections of the four groups (control and sitagliptin groups: 100, 250 and 500 mg/kg). D. Intima-media ratios (IMRs) in the three groups (n  =  10 in each group). Treatment with sitagliptin produced a lower IMR than controls in a dose-dependent manner (the lower IMR with the higher dose of sitagliptin, p < 0.05 between control and 250 mg/kg or 500 mg/kg of sitagliptin groups).

**(5) MMP2 and MMP9 expression in HUVEC.** Harvested cells were solubilized in cell lysis buffer (Cell Signaling). Protein lysate concentrations were measured using a Bradford protein assay kit (BioRad). Primary antibodies for MMP2 and MMP9 (both from Santa Cruz) were applied.

**(6) Mechanistic experiment with small interfering RNA (siRNA) against DPP-IV and CARD11.** Recently, Ohnuma et al. have shown that DPP-IV binding to CARD11 induces NFκB activation in T-cells [20]. To determine whether both of DPP-IV and CARD11 can affect NFκB activation in VSMCs, we examined the effect of DPP-IV and CARD11 knockdown in VSMCs. Presence of DPP-IV enzyme in VSMCs was identified by RT-PCR. PDGF-BB and TNFα were used to check their effects on DPP-IV expression. For knockdown of DPP-IV, VSMCs were transfected with siDPP-IV (Dharmacon, 50nM) using Lipofectamine RNAiMAX (Invitrogen). Thymidine incorporation assay was performed with siDPP-IV to confirm that direct inhibition of DPP-IV was involved in attenuated cell proliferation which was induced by FBS or PDGF-BB. Changes of NFκB activation by siDPP-IV or sitagliptin were also evaluated in EMSA study. The effect of sitagliptin on the expression of CARD11 by RT-PCR in VSMCs was also explored. For knockdown of CARD11, VSMCs were transfected with siCARD11 (Dharmacon, 50 nM) using Lipofectamine RNAiMAX. Similarly, to investigate involvement of CARD11 in NFκB pathway, changes of NFκB activation by siCARD11 was determined by EMSA study. Detailed information of RT-PCR and EMSA was described in the online supplement.

### Statistical Analysis

Results are reported as means ± SD. Mean values are compared for the DPP-IV inhibitor treated group and the control group by ANOVA with the post hoc test, and p < 0.05 is considered statistically significant (SPSS 12.0, Chicago, IL).

**Table 1:** **Weight, biochemical parameters including glucose homeostasis, lipids, adipocytokines, and inflammatory markers according to the treatment groups.**

	**Control**	**Sita100**	**Sita250**	**Sita500**	**P** †	**Post hoc** ‡				
Weight (g)	588.4	27.4	590.0	32.7	585.8	35.5	597.0	28.4	NS	
Fasting glucose (mg/dl)	137.8	25.1	137.3	21.7	126.8	25.9	109.3	15.2	0.069	C,E
Postload glucose (mg/dl)	197.2	26.0	186.4	29.2	184.9	16.2	159.4	16.7	0.022	C,E,F
AUC_glucose_	579.9	45.0	563.9	42.8	536.1	37.4	521.9	40.4	0.035	B,C
Insulin (pg/ml)	108.6	50.8	119.4	61.0	90.2	40.1	97.1	10.4	NS	
HOMA-IR	38.4	22.6	42.3	27.0	27.7	11.7	26.4	6.3	NS	
HOMA-B	535.4	214.3	571.6	209.3	578.6	296.1	796.4	151.7	NS	C
QUICKI	0.15	0.01	0.15	0.01	0.16	0.01	0.16	0.01	NS	
Total C (mg/dl)	77.4	11.2	88.0	21.5	72.1	12.3	81.2	22.2	NS	
Triglycerides (mg/dl)	92.7	32.9	90.5	23.0	66.3	27.1	70.2	35.3	NS	
HDL-C (mg/dl)	24.7	3.0	26.1	5.7	23.4	7.9	27.8	7.1	NS	
LDL-C (mg/dl)	35.4	5.2	43.8	14.1	35.5	6.2	37.4	7.0	NS	
Adiponectin (µg/ml)	8.0	1.7	9.1	3.2	10.3	2.6	12.5	3.7	0.021	C,E
HsCRP (mg/l)	0.19	0.06	0.17	0.05	0.15	0.06	0.12	0.05	0.093	C
TNFα (pg/ml)	5.8	2.0	5.2	1.7	4.6	1.7	4.2	1.9	NS	
IL6 (pg/ml)	12.1	5.4	13.3	6.0	11.1	4.3	10.1	2.9	NS	
MCP-1 (pg/ml)	162.1	61.6	144.8	37.1	115.8	39.6	107.7	32.1	0.073	B,C
PAI-1 (pg/ml)	224.4	72.0	263.8	96.9	187.8	83.6	171.9	52.8	NS	E

Data are means±S.D, Key: sita, sitagliptin; AUCglucose, Area under the curve of glucose; HOMA-IR and HOMA-B, homeostasis model assessment of insulin resistance and β-cell function; QUICKI, quantitative insulin check index;

† Statistical significance by oneway analysis of variances among groups.

‡ Post hoc analysis by least significant difference t test (mean difference between two groups: A  =  Control vs. sita100, B  =  Control vs. sita250, C  =  Control vs. sita500, D  =  sita100 vs. sita250, E  =  sita100 vs. sita500, F  =  sita250 vs. sita500, P < 0.05 in all cases).

## Results

### Animal Study

**(1) Biochemical parameters including plasma active GLP-1 and glucagon levels, glucose metabolism, adiponectin and inflammatory status after 3 week treatment of sitagliptin.** Plasma active GLP-1 and glucagon levels were measured at 4 hour post dosing after 3 week treatment of sitagliptin. Plasma active GLP-1 levels increased and glucagon levels decreased significantly in a dose-dependent manner (*p < 0.05 compared with control and †p < 0.05 compared with 100 mg/kg of sitagliptin treatment) (**Fig. 1A and 1B**).

Three week treatment of sitagliptin reduced postprandial glucose level significantly and dose dependently, although there was borderline significance in reduction of fasting glucose level by sitagliptin treatment (**Table 1**). There was no difference in IPGTT findings at baseline (data not shown). However, after 3 weeks treatment of sitagliptin, glucose tolerance was improved in these animals, as indicated by the significantly decreased AUC_glucose_ during OGTT in the DPP-IV inhibitor-treated rats (500 mg/kg of sitagliptin) as compared to the control rats (**Table 1**). HOMA-B, which is a surrogate marker of beta cell function of pancreas, was increased significantly higher in high dose treatment of sitagliptin than control. Furthermore, the treatment with sitagliptin induced increasing pattern of plasma concentration of adiponectin, which is known to be closely associated with insulin sensitivity. Plasma levels of hsCRP and MCP-1, well known inflammatory markers, were decreased by the treatment of DPP-IV inhibitors although statistical significance was only found between high dose of sitagliptin groups and control rat (**Table 1**). Other inflammatory or thrombotic markers (TNFα, IL6 or PAI-1) were also found to have decreasing patterns by sitagliptin treatment although there was no statistical significance. Furthermore, there were significant correlations between inflammatory or thrombotic markers and IMR (**Fig. S1**)**.**

**Figure 2:**
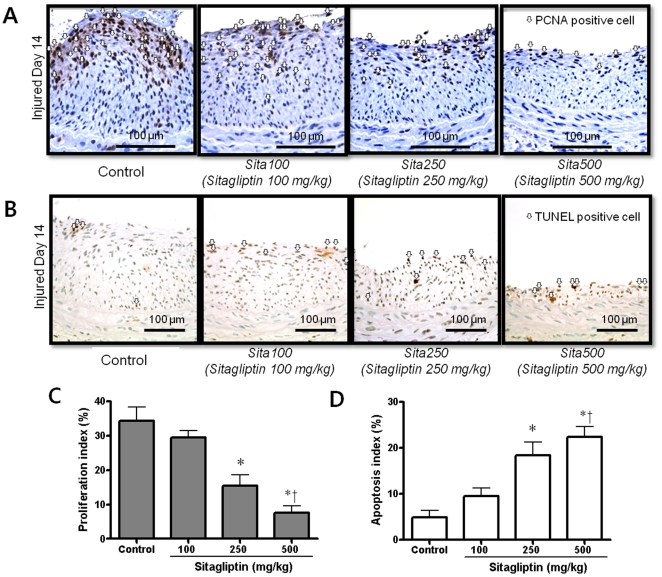
Effects of sitagliptin treatment or normal saline as a control on proliferation and apoptosis of vascular smooth muscle cells. A. Cell proliferation measured by immunostaining for proliferating cell nuclear antigen (PCNA) was markedly lower in the sitagliptin-treated groups than in the controls (open arrow). B. TUNEL staining of the three groups (open arrow). C. The proliferation index was significantly lower in the sitagliptin-treated groups than in the control group. There was a dose-dependent pattern in the level of proliferation between sitagliptin-treated groups (*p < 0.05 vs. control and †p < 0.05 vs. sitagliptin 100 mg/kg). D. Apoptosis index (%) at 2 weeks after balloon injury. Apoptosis was significantly higher in the sitagliptin -treated groups than in the control group, and there was a dose-dependent pattern in the level of apoptosis between sitagliptin-treated groups (*p < 0.05 vs. control and †p < 0.05 vs. sitagliptin 100 mg/kg).

**Figure 3:**
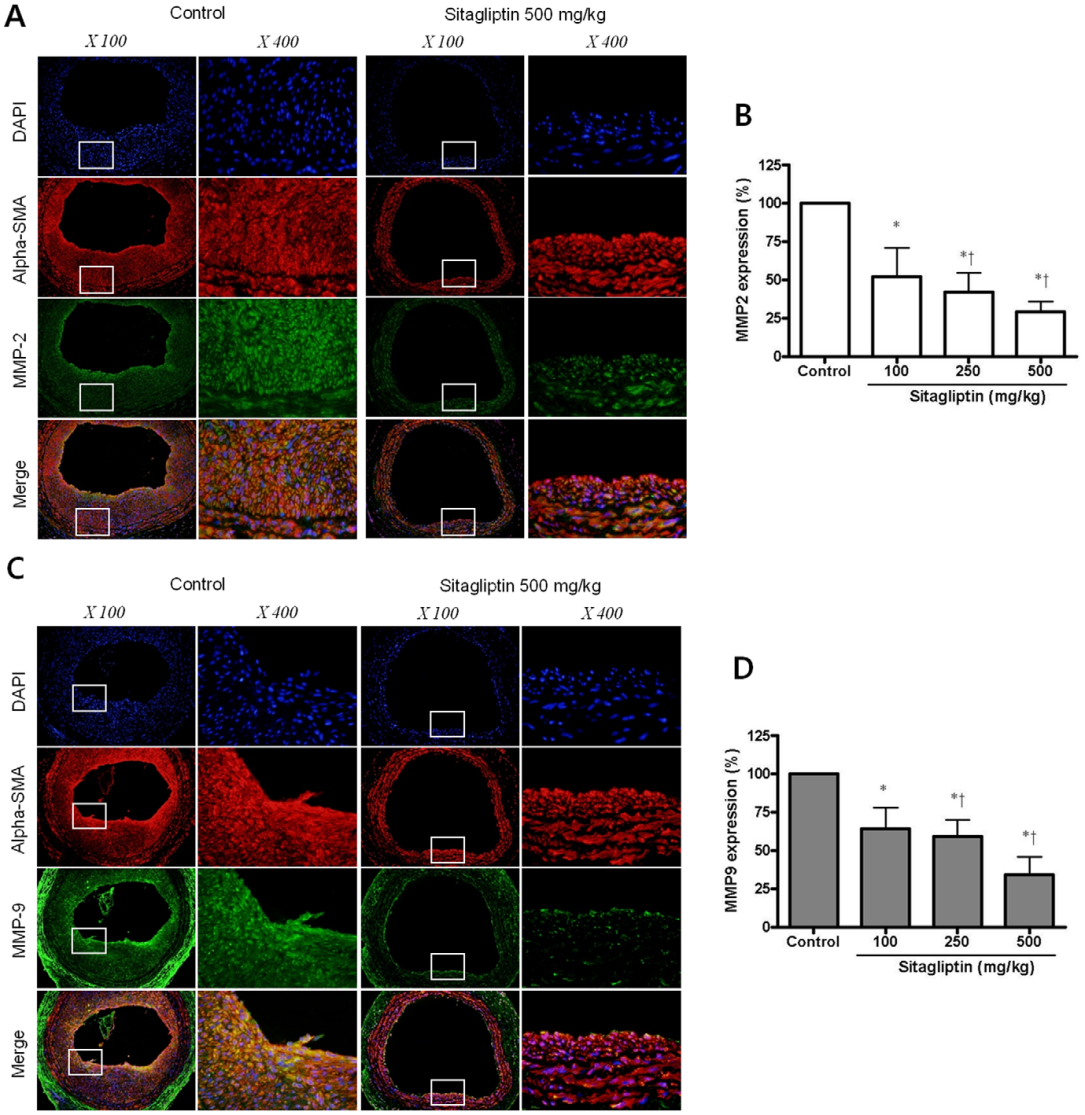
Immunofluorescent double staining for MMP2 (A) and MMP9 (C) expressing cells in tissue sections of injured arteries. Double-staining was done with the anti-αSMA antibody (red) and anti-MMP2 or MMP9 antibody (green). Nucleus of cells was stained by DAPI (blue). MMP2 and MMP9 expressions were reduced by sitagliptin treatment (500 mg/kg) compared to control. Two scaled photos were displayed (×100 and ×400). Effect of sitagliptin on MMP2 (B) and MMP9 (D) expression in tissue sections of injured arteries (*p < 0.01 compared with control and †p < 0.01 compared with 100 mg/kg of sitagliptin treatment).

**Figure 4:**
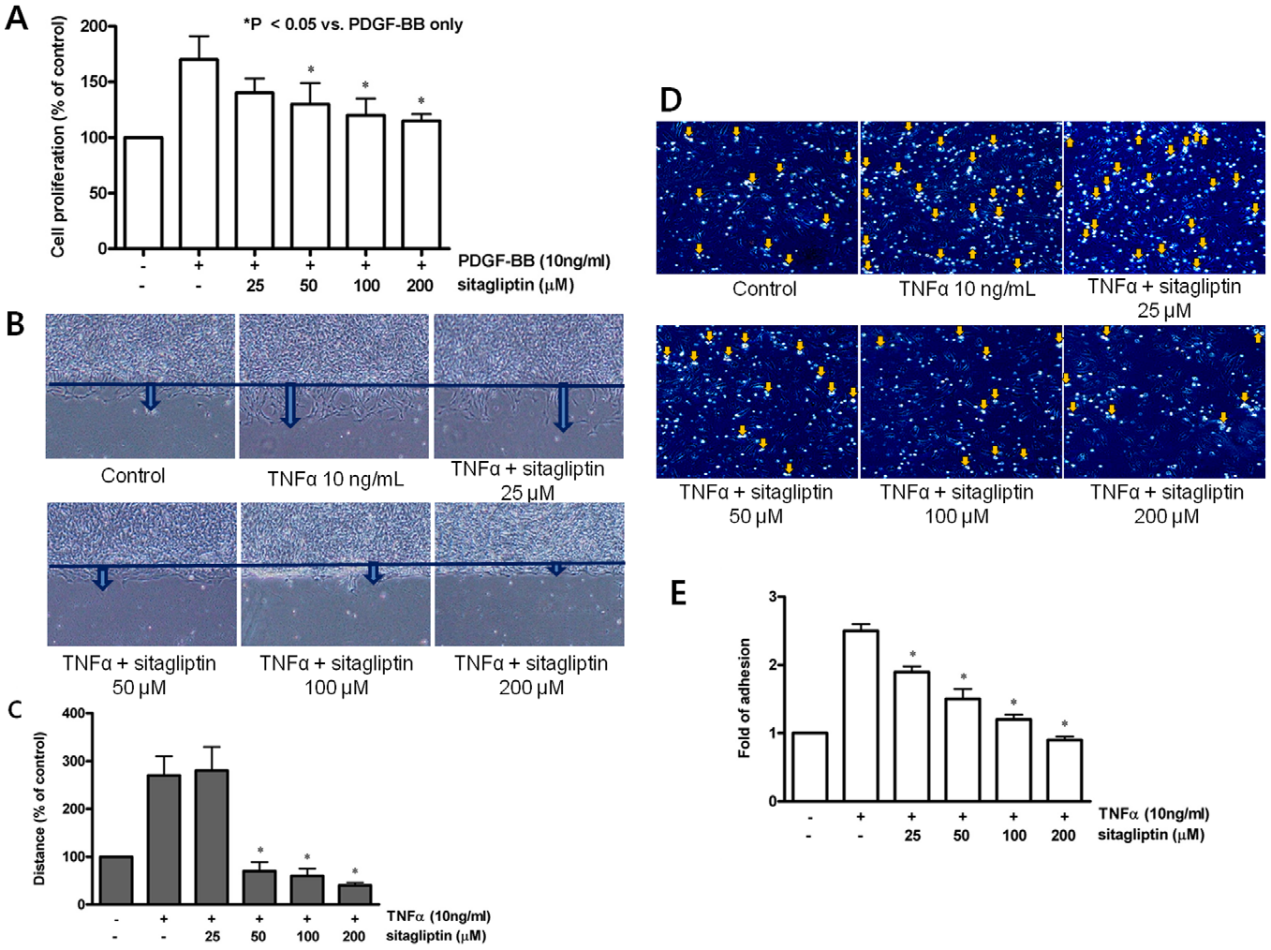
Effects of sitagliptin on proliferation and migration of rat aortic smooth muscle cells (RAoSMC). A. In MTT viability assays, cell proliferation was significantly decreased by sitagliptin treatment (*p < 0.05 compared with PDGF-BB only). B**.** TNFα-directed migration with sitagliptin treatment. C. Dose-dependent inhibiting pattern of TNFα-directed migration from 50 to 200 µg/mL (*p < 0.05 compared with TNFα treatment). D. Effects of sitagliptin on TNFα-stimulated monocyte adhesion using U937 cells. Open arrows indicate adhered monocytes. E. Dose-dependent inhibiting pattern of TNFα-stimulated monocyte adhesion (*p < 0.05 compared with TNFα treatment).

**Figure 5:**
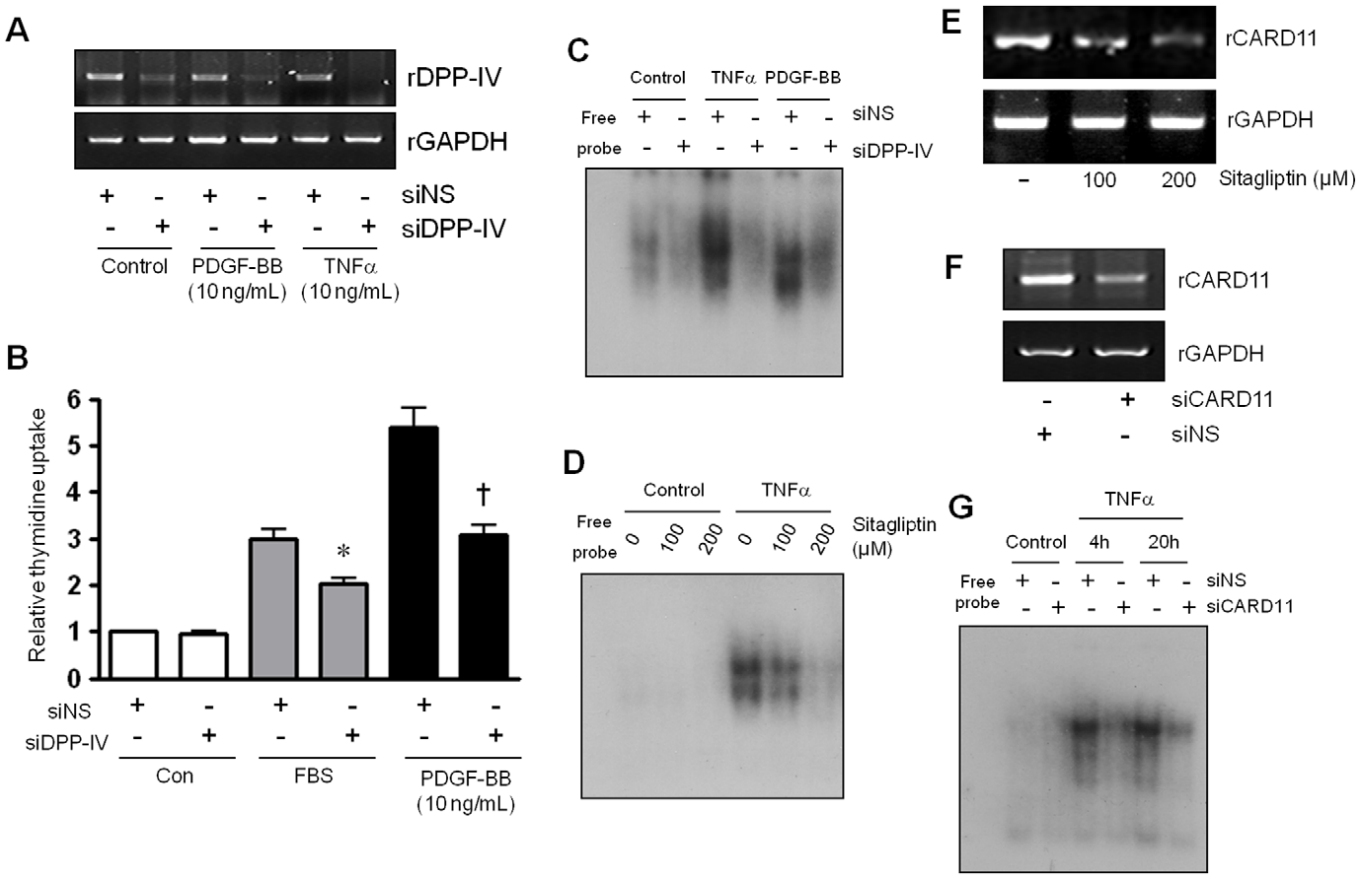
Mechanistic experiment with small interfering RNA (siRNA) against DPP-IV and CARD11. A. DPP-IV expression in VSMCs. The DPP-IV expression was knockdowned by siDPP-IV while it was not changed by treatment of PDGF-BB or TNFα. B. FBS- or PDGF-BB-induced cell proliferation was significantly attenuated by siDPP-IV transfection. C. Attenuation of TNFα- or PDGF-BB-induced NFκB activation by siDPP-IV transfection was also confirmed in EMSA assay. D. TNFα-induced NFκB activation was attenuated by sitagliptin treatment dose-dependently. E. Expression of CARD11 was decreased dose-dependently by sitagliptin treatment in VSMCs. F. CARD11 was expressed in VSMCs and its expression was knockdowned by siCARD11. G. TNFα-induced NFκB activation was decreased by siCARD11 transfection in EMSA assay.

**(2) In vivo inhibition of neointimal formation.** To determine whether sitagliptin treatment can alter neointimal formation, we measured the IMR in H&E stained tissue sections of injured arteries in OLETF rats. Two weeks after injury, sitagliptin treated groups (sitagliptin 250 mg and 500 mg) showed a significant reduction in neointimal formation versus the control group (**Fig. 1C**). As shown in **Fig. 1D**, there was a dose-dependent relationship in the reduction of IMR among the sitagliptin treated groups (p < 0.05). A similar but slightly attenuated result was found in LETO rats (**Fig. S2**)**.**

**(3) DPP-IV and GLP-1 receptor expression in the carotid artery.** The expression of DPP-IV and the GLP-1 receptor was confirmed by Western blotting of the harvested vessel tissues (**Fig. S3A**). The expression of DPP-IV and the GLP-1 receptors did not differ significantly between the control and sitagliptin groups (**Fig. S3B and S3C**).

**(4) Inhibited proliferation and sustained apoptosis of vascular smooth muscle cells after sitagliptin treatment.** To determine whether sitagliptin can affect cell proliferation directly, we measured cell proliferation by immunostaining for PCNA in the 5 tissue sections of injured arteries. As shown in **Fig. 2A**, cell proliferation was markedly lower in the sitagliptin-treated groups than in the controls (open arrow). There was a dose-dependently decreasing pattern in the proliferation index among sitagliptin treated groups (Control, 34.2 ± 4.1%; sitagliptin 100 mg, 29.4 ± 2.0%; sitagliptin 250 mg, 15.4 ± 3.2%; sitagliptin 500 mg, 7.6 ± 2.1%, *P for trend* < 0.01) (**Fig. 2C**).

To examine the effects of sitagliptin treatment on apoptosis after balloon injury *in vivo,* we performed TUNEL staining in the 5 tissue sections of injured arteries (**Fig. 2B**). After three weeks treatment of sitagliptin (at two weeks after balloon injury), the apoptosis index was increased significantly in the sitagliptin groups compared to the control group, which was in a dose-dependent manner among sitagliptin treated groups (9.5 ± 1.7% in sitagliptin 100 mg, 18.4 ± 2.9% in sitagliptin 250 mg and 22.4 ± 2.2% in sitagliptin 500 mg compared to control, *P for trend* < 0.01) (**Fig. 2D**). Using double staining for α-smooth muscle actin and TUNEL, we identified the apoptotic cells as smooth muscle cells; there were more vascular smooth muscle cell apoptotic bodies in the sitagliptin treated group than in control group (p < 0.01) (**Fig. S4**).

**(5) Decrease in MMP2 and MMP9 expression in sitagliptin treatment groups.** To clarify whether proliferating smooth muscle cells correspond to the cells expressing MMP2, we performed double-staining with the anti-αSMA antibody (red) and anti-MMP2 antibody (green) (**Fig. 3A**) or anti-MMP9 antibody (green) (**Fig. 3C**) in injured arteries. Nucleus of cells was stained by DAPI (blue). Fewer MMP2-expressing cells (**Fig. 3B**) and MMP9-expressing cells (**Fig. 3D**) were observed in the sitagliptin treated-groups than in the control groups, and the effects were dose-dependent.

### Cell Study

**(1) Proliferation and migration of VSMCs and monocyte adhesion in vitro after sitagliptin treatment.** In MTT assays, sitagliptin treatment inhibited PDGF-BB-induced cell proliferation in RAoSMCs (**Fig. 4A**). This effect was initiated at the dose of 50 µM and was increased dose dependently to be more prominent at the dose of 200 µM. Similar finding was found in the thymidine incorporation assay (**Fig. S5**). Sitagliptin treatment also inhibited TNFα-directed migration of RAoSMCs in a dose-dependent manner from 50 to 200 µg/mL (**Fig. 4B, 4C**). To test whether sitagliptin can prevent TNFα-induced monocyte adhesion, we performed the monocyte adhesion assay by using U937 cells. TNFα treatment significantly increased monocyte adhesion in U937 cells, and these effects were blocked by treatment of sitagliptin in a dose-dependent manner (**Fig. 4D, 4E**). This antiproliferative property of sitagliptin was not associated with cytotoxicity, as shown by the calcein measurement and WST-8 assay (**Fig. S6A and S6B**).

**(2) Change of caspase-3 activity after sitagliptin treatment.** Apoptosis, a type of programmed cell death, is one method of controlling immune responses such as cellular homeostasis as well as a variety of physiological processes. Caspases, a family of cysteine proteases, play a central role in the apoptosis [24]. TNFα is secreted by VSMCs in the neointima after a balloon injury as well as by macrophages in atherosclerotic lesions [25]. Therefore, the effect of sitagliptin treatment on caspase-3 activity was investigated in TNFα-stimulated VSMCs. Sitagliptin treatment increased the level of caspase-3 activity in a dose-dependent manner, reflecting apoptosis in RAoSMCs (**Fig. S7**).

**(3) Effect of sitagliptin on MMP2 and MMP9 expression in HUVECs and VSMCs.** Increased proteolytic activity in the vessel wall mediates the degradation of the ECM surrounding smooth muscle cells in response to injury [26], a necessary step to allow medial smooth muscle cells to migrate into the intimal space. Matrix metalloproteinases (MMPs) such as MMP2 (72 kDa) and MMP9 (92 kDa), have been implicated as mediator of lesion development in response to vascular injury [27], [28]. The effect of sitagliptin on MMP2 and MMP9 expression was examined in HUVECs and VSMCs. Expression of MMP2 and MMP9 decreased significantly in a dose-dependent manner after treatment with sitagliptin compared with TNFα treatment (p < 0.01) (**Fig. S8 and Fig. S9**).

**(4) Effect of DPP-IV and CARD11 knockdown in VSMCs.** DPP-IV was expressed in VSMCs and was knockdowned by siDPP-IV (**Fig. 5A**). The expression of DPP-IV was not changed by treatment of PDGF-BB or TNFα. FBS- or PDGF-BB-induced cell proliferation was significantly attenuated by siDPP-IV transfection (**Fig. 5B**). Attenuation of TNFα- or PDGF-BB-induced NFκB activation by siDPP-IV transfection was also confirmed in EMSA assay (**Fig. 5C**). TNFα-induced NFκB activation was attenuated by sitagliptin treatment dose-dependently (**Fig. 5D**). Expression of CARD11 was decreased dose-dependently by sitagliptin treatment in VSMCs (**Fig. 5E**). CARD11 was expressed in VSMCs and its expression was knockdowned by siCard11 (**Fig. 5F**). TNFα-induced NFκB activation was decreased by siCARD11 transfection in EMSA assay (**Fig. 5G**).

## Discussion

The current study demonstrated that treatment with des-fluoro-sitagliptin, a DPP-IV inhibitor, reduced restenosis in obese type 2 diabetic rats following balloon injury to the carotid artery. In vivo and in vitro studies revealed that sitagliptin treatment suppressed proliferation of VSMCs, promoted apoptosis and reduced inflammatory process. Sitagliptin therapy also reduced hsCRP and MCP-1 levels and increased adiponectin concentration, indicating decrease of markers of inflammation and procoagulation, and increase of insulin sensitivity. These findings suggest that sitagliptin has protective role of development of macrovascular complication, supporting an emerging role of sitagliptin in modifying the elevated cardiovascular risks that are inherent in obesity and type 2 diabetes.

Interestingly, it was recently demonstrated that GLP-1 ameliorates endothelial dysfunction in type 2 diabetes mellitus patients with established coronary heart disease, suggesting a new important cardioprotective role for GLP-1 [29], [30]. In other study, GLP-1 infusion significantly increased relative changes in brachial artery diameter from baseline flow mediated dilatation (%) (3.1 ± 0.6 vs. 6.6 ± 1.0%, *p* < 0.05) in type 2 diabetes patients with coronary artery disease [29], just as GLP-1 increased intracellular cAMP (from 5.7 ± 0.5 to 13.1 ± 0.12 pmol/mg protein) in rat cardiac myocytes [31]. Considering inhibition of DPP-IV has been demonstrated to increase concentrations of intact GLP-1 2–3 fold in patients with type 2 diabetes [3], it is conceivable that sitagliptin, a potent and highly selective DPP-IV inhibitor, improves endothelial dysfunction as a result.

In addition, it is well known that infiltration of inflammatory cells occurs early after endothelial denudation [32]–[35]. Inhibition of this process has been reported to be associated with a reduction in medial VSMC proliferation [33]. Considering previous studies showing linear relationship between tissue monocyte content and neointimal area [35] and decreased neointimal thickening through blocking early monocyte recruitment by anti-inflammatory drug [36], inflammatory response related with monocyte infiltration could be a causative factor in restenosis.

In the current study, sitagliptin treatment also reduced MMP2 and MMP9 expression in tissue sections in injured arteries. The MMPs are a family of molecules that are associated with the breakdown of constituents of the ECM. Both MMPs and their tissue inhibitors are involved in the regulation of the ECM metabolism [37]. ECM is a dynamic structure that requires constant synthesis and degradation by MMPs [38]. Either MMP2 or MMP9 are synthesized and secreted locally in atherosclerotic lesions, predominantly by monocyte-derived macrophages and endothelial cells [39] and may participate in rupture of the atherosclerotic plaque [40]. Type 2 diabetic patients are at high risk for acute coronary events due to an increased propensity of their atherosclerotic plaques to ulceration and resultant overlying thrombosis [41], and have increased level of MMP2 and MMP9 [14]. Thus, as shown in our study, the reduction of MMP2 and MMP9 may be a possible mechanism in preventing macrovascular complications in patients with type 2 diabetes by sitagliptin treatment.

MCP-1, which is an important factor of monocyte recruitment, has been shown to play a pivotal role in the development of atherosclerosis and involves a sequence of events that include monocyte attraction, tethering and rolling, and firm adhesion [36], [42]. Recently it has been described that blocking of the MCP-1 pathway results in reduced atherosclerosis and restenosis by inhibition of monocyte adhesion to the vascular wall and to reduced macrophage content in the atherosclerotic lesion [36]. In this study, sitagliptin treatment significantly and dose-dependently reduced the degree of monocyte adhesion and showed decreasing pattern of serum concentration of MCP-1. These findings suggest another mechanism by which sitagliptin has beneficial effect on counteracting restenosis. In addition, such infiltrations of inflammatory cells are known to promote an atherosclerotic milieu [32], [43], [44]. Whether sitagliptin has the ability to positively influence atherosclerotic plaque formation would warrant further investigation.

NFκB is a common regulator that involves in the control of proinflammatory genes and VSMCs proliferation. It has been reported that DPP-IV interacting with CARD11 or CARMA1 leads to NFκB activation in T-cells [20]. In the present study, NFκB activation was reduced by DPP-IV or CARD11 Knockdown, and attenuated dose-dependently by sitagliptin treatment in VSMCs. Proliferation of VSMCs was inhibited by siDPP-IV transfection and sitagliptin treatment. These results indicate that gene expression of DPP-IV as well as enzyme activity of DPP-IV plays a crucial role in NFκB activation. Thus, reduction of NFκB activation by siDPP-IV and sitagliptin could be a major mechanism in decrease of VSMCs proliferation.

Other possible relevant factors affecting the degree of neointimal formation were considered in this study. Glucose lowering effect by sitagliptin treatment might contribute to this but sitagliptin were treated only for 3 weeks. Circulating levels of adiponectin were increased significantly in sitagliptin treatment in a dose-dependent manner. Low adiponectin level is a risk factor for the subsequent development of cardiovascular diseases [17], [19]. Further, high dose of sitagliptin induced decreasing pattern of hsCRP and PAI-1 activity in this study although statistical significance was not obtained. This finding is in line with a recent study describing that GLP-1 treatment attenuated mRNA expression by TNFα and induction of PAI-1 protein [45]. In the latter study, GLP-1 also inhibited the effect of TNFα on a reporter gene construct harboring the proximal PAI-1 promoter.

In conclusion, this study, in addition to glucose lowering effects, demonstrates that sitagliptin, a DPP-IV inhibitor, has protective properties against restenosis after carotid injury in an animal model of type 2 diabetes and vascular cell lines. These finding raise the possibility that sitagliptin could offer a novel agent for the treatment of macrovascular-related complications in patients with type 2 diabetes.

## Supporting Information

Figure S1 **Correlations between intima-media ratio (IMR) and hsCRP, TNFα and MCP-1 levels and PAI-1 activity.** There were positive correlations between IMR and each factor (p < 0.05 except IMR vs. PAI-1 activity).
(TIF)

Figure S2 **In vivo inhibition of neointimal formation after 3 weeks of treatment with des-fluoro-sitagliptin in LETO rats.** A, H&E-stained sections of the control and sitagliptin (500 mg/kg) groups. B, Intima-media ratios (IMRs) in the two groups (n  =  10 in each group). The IMR was calculated from the mean areas of the intima and media. Treatment with sitagliptin produced a lower IMR than in controls (p < 0.05 between the control and 500 mg/kg sitagliptin-treated groups).
(TIF)

Figure S3 **A. Western blot of DPP-IV and GLP-1 receptor in the injured carotid arteries of control and sitagliptin treated rats.** Representative three samples were displayed. Quantification of Western blot images of DPP-IV (B) and GLP-1 receptor (C).
(TIF)

Figure S4 **Double staining of α-smooth muscle actin (αSMA) and TUNEL in the injured carotid vessel wall.** Apoptotic cells were smooth muscle cells; there were more vascular smooth muscle cell apoptotic bodies in the sitagliptin treated group (18.7%) than in the control group (5.2%) (p < 0.01) (Arrows indicate vascular smooth muscle cell apoptotic bodies).
(TIF)

Figure S5 **Thymidine incorporation assay to check effect of sitagliptin on FBS- or PDGF-induced cell proliferation.** There were dose-dependent decreasing patterns of thymidine uptake by sitagliptin treatment (*p < 0.05 compared with FBS or PDGF-BB treatment only).
(TIF)

Figure S6 **Effect of sitagliptin on cell survival.** A. Calcein-acetoxymethyl ester (calcein-AM) cell viability assay kit was used (Biotium, Hayward, CA, USA). Cells were washed with PBS and incubated with 2 µM calcein AM for 30 min. The fluorescence was measured using 485 nm excitation wavelength and 530 nm emission wavelength with a Victor 3 instrument (Perkin-Elmer, Boston, MA, USA). B. Cell viability was also measured with Cell Counting Kit-8 (CCK-8, Dojindo, Japan). Absorbance was measured at 450 nm (VersaMax; Molecular Devices, Sunnyvale, CA, USA). Cell Counting Kit-8 (CCK-8) allows convenient assays by utilizing Dojindo’s highly water-soluble tetrazolium salt. WST-8 [2-(2-methoxy-4-nitrophenyl)-3-(4-nitrophenyl)-5-(2,4-disulfophenyl)-2H-tetrazolium, monosodium salt] produces a water-soluble formazan dye upon reduction in the presence of an electron carrier. WST-8 is reduced by dehydrogenases in cells to give a yellow-colored product (formazan), which is soluble in the tissue culture medium. The amount of the formazan dye generated by the activity of dehydrogenases in cells is directly proportional to the number of living cells.
(TIF)

Figure S7 **Induction of apoptosis shown by the activation of caspase-3 with sitagliptin treatment in VSMCs.** There was a dose-dependent increasing pattern of caspase-3 activity (*p < 0.05 compared with TNFα treatment only).
(TIF)

Figure S8 **Effects of sitagliptin on MMP2 and MMP9 expression levels in human umbilical vein endothelial cells (A).** Expressions of MMP2 (B) and MMP9 (C) decreased significantly with the treatment of sitagliptin compared to TNFα treatment in a dose-dependent manner (*p < 0.05 compared with TNFα treatment).
(TIF)

Figure S9 **Effects of sitagliptin on MMP2 and MMP9 expression levels in vascular smooth muscle cells (A).** Expressions of MMP2 (B) and MMP9 (C) decreased significantly with the treatment of sitagliptin compared to TNFα treatment in a dose-dependent manner (*p < 0.05 compared with TNFα treatment).
(TIF)
