# Correction: Effect of a Dipeptidyl Peptidase-IV Inhibitor, Des-Fluoro-Sitagliptin, on Neointimal Formation after Balloon Injury in Rats

**DOI:** 10.1371/annotation/f0a21e28-7f3c-4b76-870e-128dd89d0e29

**Published:** 2012-05-31

**Authors:** Soo Lim, Sung Hee Choi, Hayley Shin, Bong Jun Cho, Ho Seon Park, Byung Yong Ahn, Seon Mee Kang, Ji Won Yoon, Hak Chul Jang, Young-Bum Kim, Kyong Soo Park

The wrong author is labelled as the corresponding author. The correct corresponding author is Kyong Soo Park.

