# Supplementary figures and images for: Effect of a Dipeptidyl Peptidase-IV Inhibitor, Des-Fluoro-Sitagliptin, on Neointimal Formation after Balloon Injury in Rats
Source: PLoS One. 2012 Apr 6;7(4):e35007. doi: 10.1371/journal.pone.0035007 (PMC3320861; doi:10.1371/journal.pone.0035007)

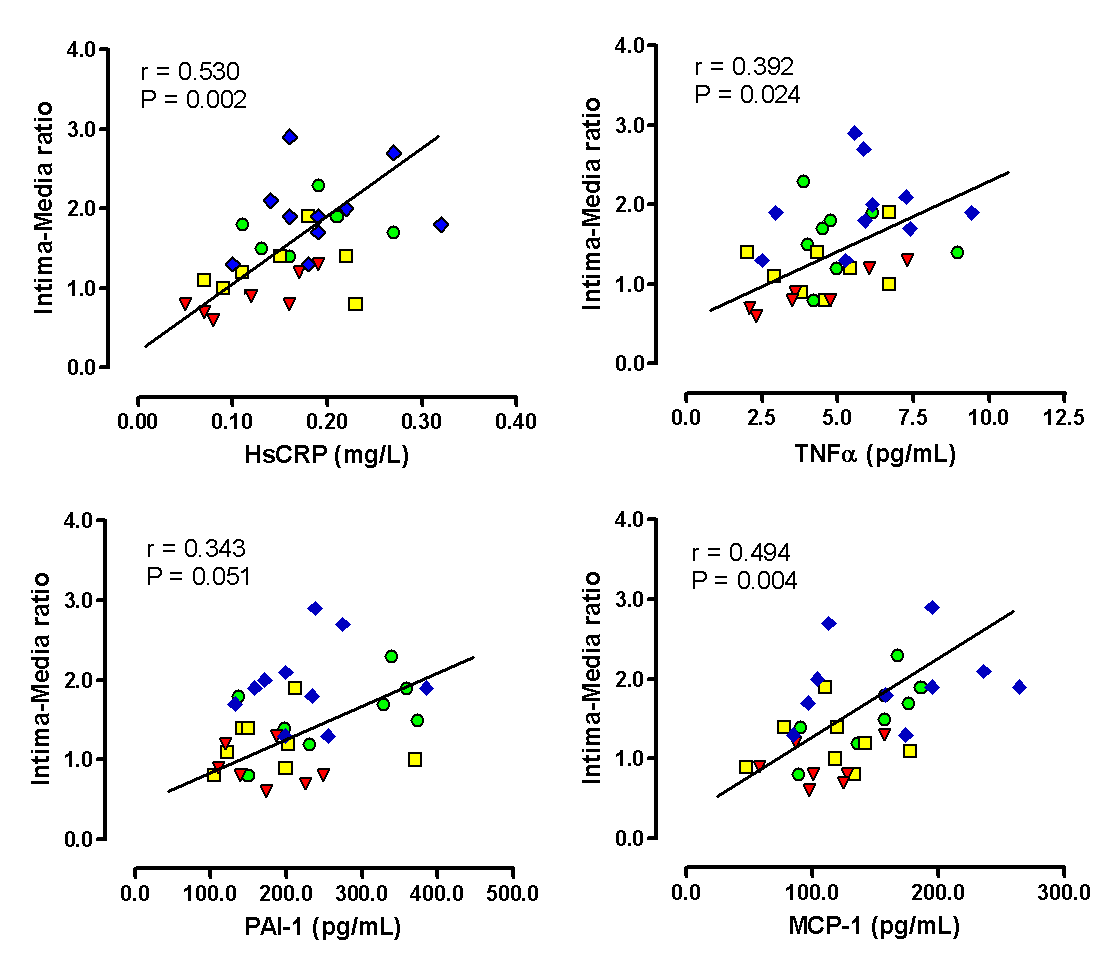

Supplement: Figure S1 — Correlations between intima-media ratio (IMR) and hsCRP, TNFα and MCP-1 levels and PAI-1 activity. There were positive correlations between IMR and each factor (p < 0.05 except IMR vs. PAI-1 activity). (TIF) [file pone.0035007.s001.tif]

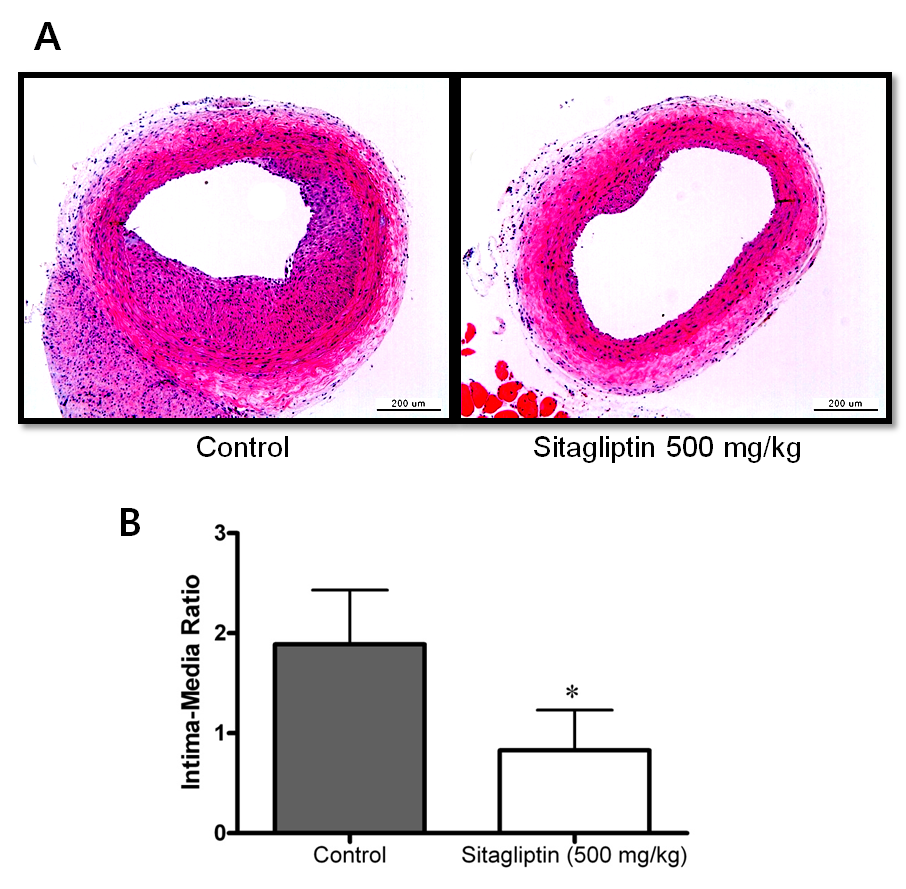

Supplement: Figure S2 — In vivo inhibition of neointimal formation after 3 weeks of treatment with des-fluoro-sitagliptin in LETO rats. A, H&E-stained sections of the control and sitagliptin (500 mg/kg) groups. B, Intima-media ratios (IMRs) in the two groups (n = 10 in each group). The IMR was calculated from the mean areas of the intima and media. Treatment with sitagliptin produced a lower IMR than in controls (p < 0.05 between the control and 500 mg/kg sitagliptin-treated groups). (TIF) [file pone.0035007.s002.tif]

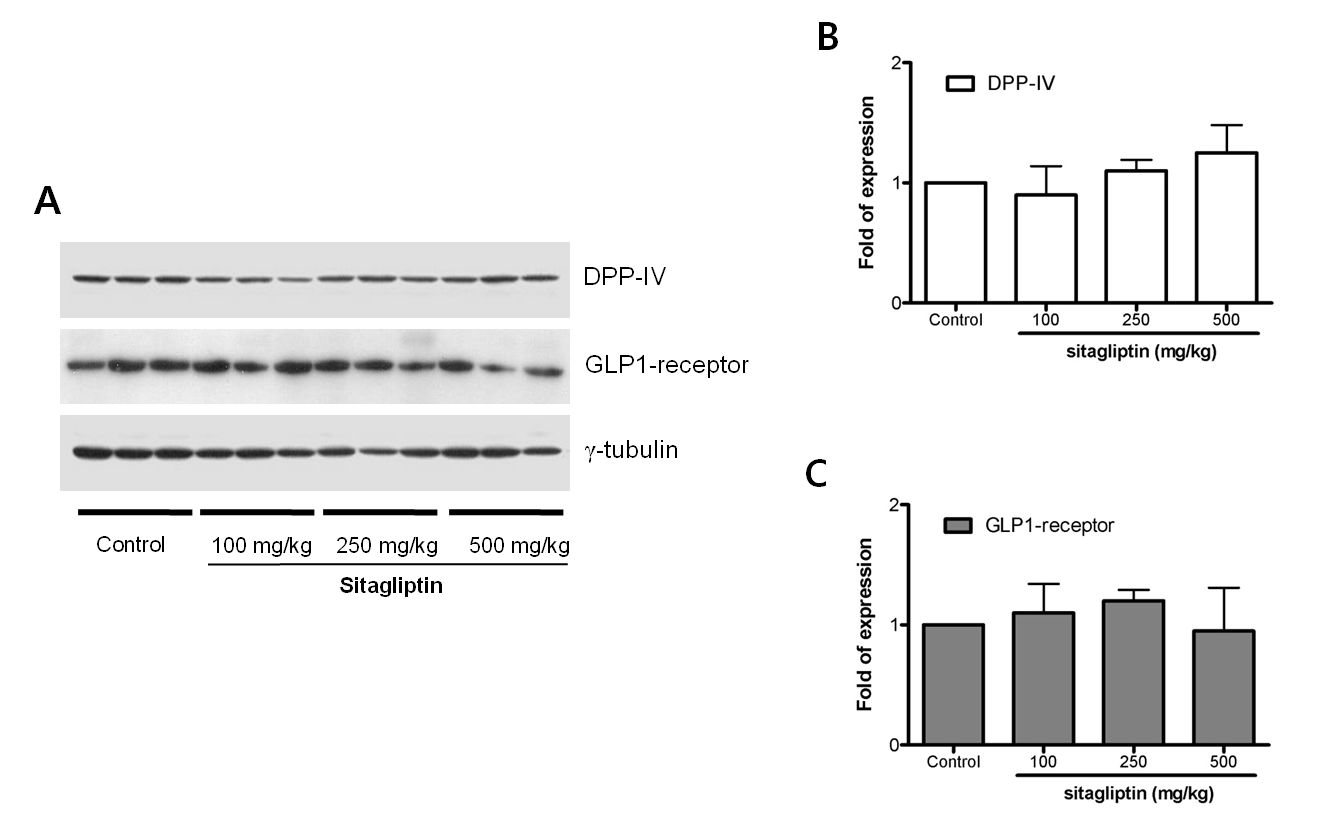

Supplement: Figure S3 — A. Western blot of DPP-IV and GLP-1 receptor in the injured carotid arteries of control and sitagliptin treated rats. Representative three samples were displayed. Quantification of Western blot images of DPP-IV (B) and GLP-1 receptor (C). (TIF) [file pone.0035007.s003.tif]

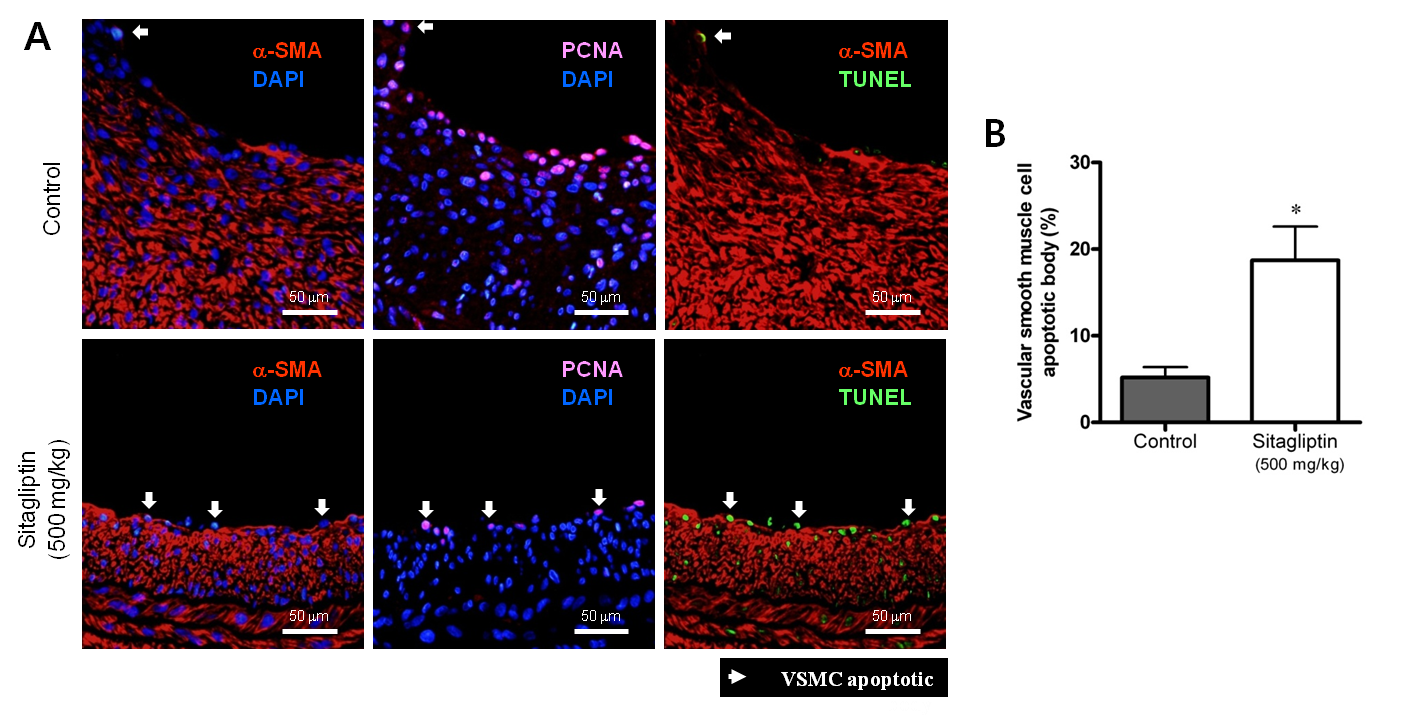

Supplement: Figure S4 — Double staining of α-smooth muscle actin (αSMA) and TUNEL in the injured carotid vessel wall. Apoptotic cells were smooth muscle cells; there were more vascular smooth muscle cell apoptotic bodies in the sitagliptin treated group (18.7%) than in the control group (5.2%) (p < 0.01) (Arrows indicate vascular smooth muscle cell apoptotic bodies). (TIF) [file pone.0035007.s004.tif]

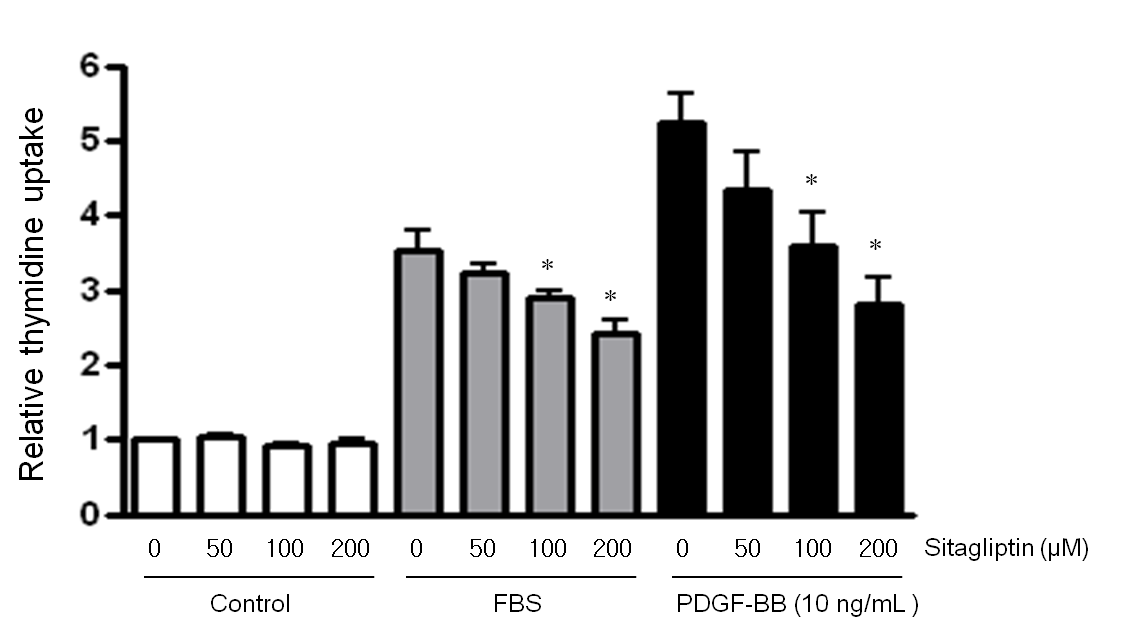

Supplement: Figure S5 — Thymidine incorporation assay to check effect of sitagliptin on FBS- or PDGF-induced cell proliferation. There were dose-dependent decreasing patterns of thymidine uptake by sitagliptin treatment (*p < 0.05 compared with FBS or PDGF-BB treatment only). (TIF) [file pone.0035007.s005.tif]

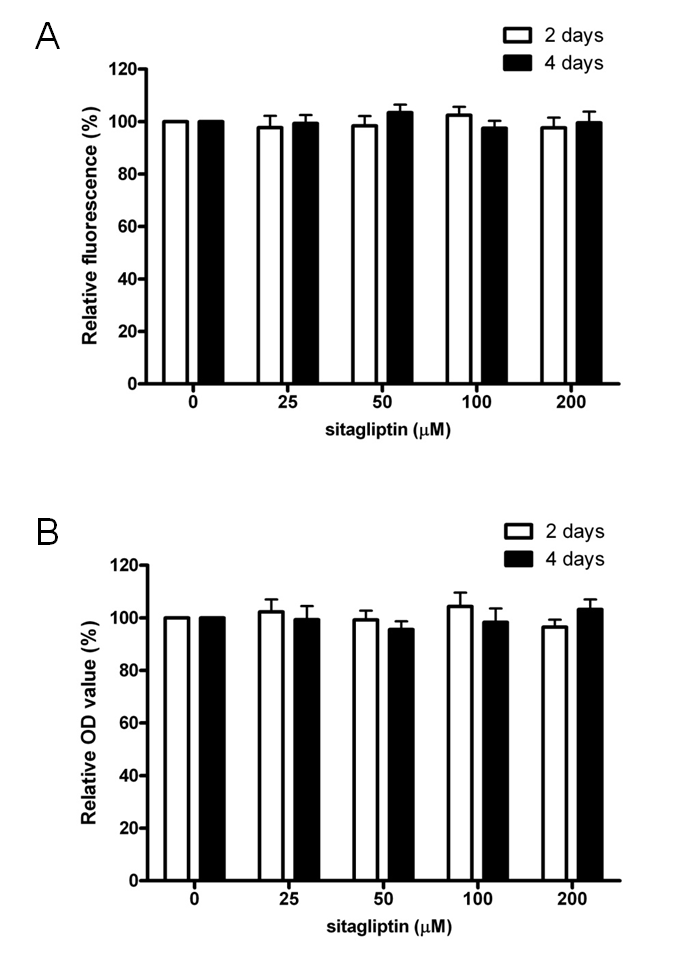

Supplement: Figure S6 — Effect of sitagliptin on cell survival. A. Calcein-acetoxymethyl ester (calcein-AM) cell viability assay kit was used (Biotium, Hayward, CA, USA). Cells were washed with PBS and incubated with 2 µM calcein AM for 30 min. The fluorescence was measured using 485 nm excitation wavelength and 530 nm emission wavelength with a Victor 3 instrument (Perkin-Elmer, Boston, MA, USA). B. Cell viability was also measured with Cell Counting Kit-8 (CCK-8, Dojindo, Japan). Absorbance was measured at 450 nm (VersaMax; Molecular Devices, Sunnyvale, CA, USA). Cell Counting Kit-8 (CCK-8) allows convenient assays by utilizing Dojindo’s highly water-soluble tetrazolium salt. WST-8 [2-(2-methoxy-4-nitrophenyl)-3-(4-nitrophenyl)-5-(2,4-disulfophenyl)-2H-tetrazolium, monosodium salt] produces a water-soluble formazan dye upon reduction in the presence of an electron carrier. WST-8 is reduced by dehydrogenases in cells to give a yellow-colored product (formazan), which is soluble in the tissue culture medium. The amount of the formazan dye generated by the activity of dehydrogenases in cells is directly proportional to the number of living cells. (TIF) [file pone.0035007.s006.tif]

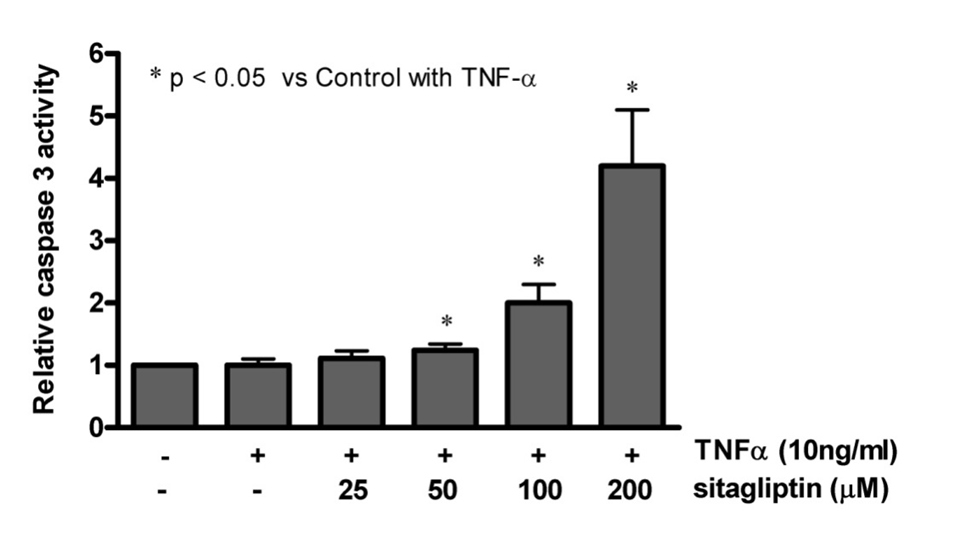

Supplement: Figure S7 — Induction of apoptosis shown by the activation of caspase-3 with sitagliptin treatment in VSMCs. There was a dose-dependent increasing pattern of caspase-3 activity (*p < 0.05 compared with TNFα treatment only). (TIF) [file pone.0035007.s007.tif]

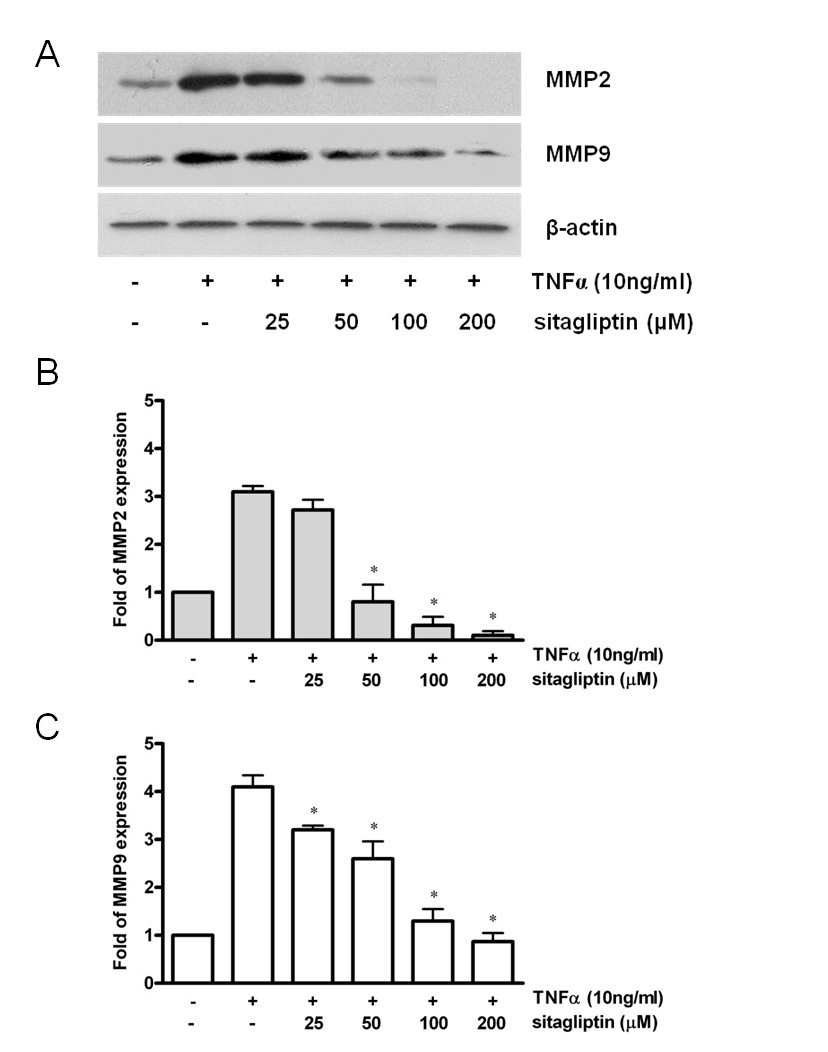

Supplement: Figure S8 — Effects of sitagliptin on MMP2 and MMP9 expression levels in human umbilical vein endothelial cells (A). Expressions of MMP2 (B) and MMP9 (C) decreased significantly with the treatment of sitagliptin compared to TNFα treatment in a dose-dependent manner (*p < 0.05 compared with TNFα treatment). (TIF) [file pone.0035007.s008.tif]

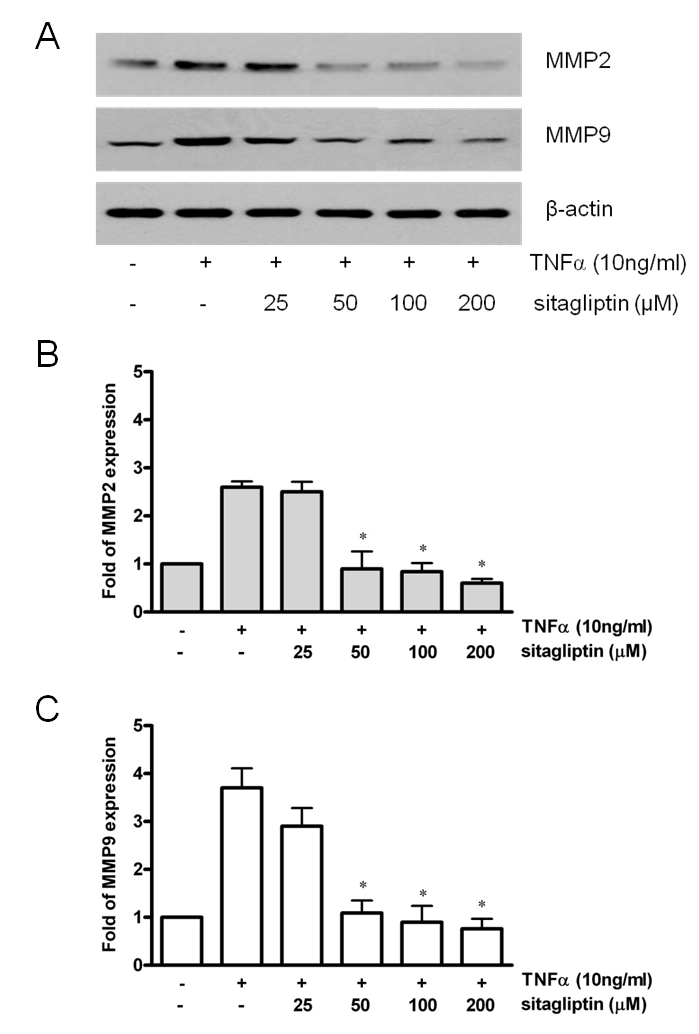

Supplement: Figure S9 — Effects of sitagliptin on MMP2 and MMP9 expression levels in vascular smooth muscle cells (A). Expressions of MMP2 (B) and MMP9 (C) decreased significantly with the treatment of sitagliptin compared to TNFα treatment in a dose-dependent manner (*p < 0.05 compared with TNFα treatment). (TIF) [file pone.0035007.s009.tif]
